# Body Mass Index versus Other Adiposity Traits: Best Predictor of Cardiometabolic Risk

**Published:** 2019-12

**Authors:** Muhammad SAQLAIN, Zainab AKHTAR, Raheela KARAMAT, Samra MUNAWAR, Maria IQBAL, Muhammad FIAZ, Muhammad Mubeen ZAFAR, Sadia SAEED, Muhammad Farooq NASIR, S.M. Saqlan NAQVI, Ghazala Kaukab RAJA

**Affiliations:** 1. Department of Biochemistry, PMAS–Arid Agriculture University, Rawalpindi, Pakistan; 2. Department of Pathology, Shaheed Zulfiqar Ali Bhutto Medical University, Islamabad, Pakistan; 3. Department of Entomology, PMAS–Arid Agriculture University, Rawalpindi, Pakistan

**Keywords:** Obesity, Body mass index, Waist circumference, Pakistan

## Abstract

**Background:**

A number of anthropometric indices have been used in different world populations as markers to estimate obesity and its related health risks. The present study is large population based study dealing with five anthropometric obesity scales; Body mass index (BMI), waist circumference (WC), waist to hip ratio (WHR), basal adiposity index (BAI), and Visceral adiposity index (VAI) to identify common adiposity trait(s) that best predict obesity and associated health complication(s).

**Methods:**

A total of 4000 subjects including 1000 in each category of BMI from four provinces (Punjab, Sindh, Kahyber pakhtoonkha and Balochistan) of Pakistan from 2012–2017 were collected. Complete anthropometric measurementswere obtained and blood samples were collected and Biochemical profiling was performed. Descriptive statistics, linear regression, binary and multiple regression analysis was done.

**Results:**

Our data analysis explored the relationships of obesity five indices; BMI, WC, WHR, BAI, and VAI with common metabolic health complications. Effect size analysis clearly indicates that a unit increase in BMI significant raised all anthropometric and clinical parameters. General and sex specific association analysis of adiposity traits with risk phenotypes (hypertension, hyperglycemia and dyslipidemia) indicated significant associations of WC with all three metabolic risks. Varying degrees of correlations of other adiposity traits with metabolic risks were observed. Frequency of different obesity classes among obese population group were as follows; 55.7% class I, 28.50% Class II and 15.80% Class III.

**Conclusion:**

WC is the strong predictor of obesity associated metabolic health issues in Pakistani populations. While BMI has significant increasing effect on other obesity indices like WHR, VAI and BAI.

## Introduction

Obesity has been declared as a global epidemic of twenty first century. The extra body fats carried by a person either generally distributed or abdominally are being considered one of the major root cause of non-communicable diseases (NCDs) like metabolic syndrome (MetS), CHD, T2D, hypertension, non-alcoholic fatty liver disease (NAFLD) etc. (1, 2). The major culprits, increasing the chance of gaining extra pounds, are recognized as unhealthy lifestyle; overindulgence of high caloric food along with lack of physical activity (3).

Since 1980s to 2013 the worldwide proportion of overweight and obese has increased from 28.8% to 36.9%. Almost 50% obese subjects in the world belong to ten countries including USA, China, India, Russia, Brazil, Mexico, Egypt, Germany, Pakistan, and Indonesia. For an accurate estimate of body fats/obesity status, imaging techniques are being recommended providing accurate estimates of body fat compartments (4). For large population based surveys anthropometric indices like Body Mass Index (BMI) (Weight and height ratio), waist circumferences (WC), hip circumferences (HC) and waist-hip ratio (WHR) are commonly used offering simplicity and reliability in obesity assessment (5). The international as well as ethnicity, age, and sex specific cut-off values for BMI, WC, and WHR are being calculated by the experts (5). Though BMI is one of the extensively used global anthropometric marker for the assessment of generalized obesity (6), it is generally not considered as a reliable measure of body fatness and prediction of associated health risks (7, 8). In contrast WC and WHR specifically target body shape and thus are considered more reliable estimate of the abdominal/central obesity status of an individual despite having BMI within the normal ranges (9). Two newly introduced adiposity indices are Body Adiposity Index (BAI) and Visceral Adiposity Index (VAI). BAI estimates body fat percentage using height and HC with sex and ethnicity specific cut-off values while VAI provides sex-specific visceral adiposity measure using WC, BMI, triglycerides, and HDL cholesterol (10, 11). The obesity indices BMI, WC, WHR, BAI, and VAI have been extensively explored in different world populations as risk markers of various metabolic health complications (12–15). Regardless of age and sex, the prevalence of obesity and metabolic health complications is on rise in Pakistan (16). However large population based data estimating obesity indices and their associations with major metabolic traits from Pakistani population is still lacking.

In this study we took in account the correlations of conventional anthropometric obesity traits; BMI, WC, WHR, BAI, and VAI with major metabolic risk phenotypes aimed to identify common adiposity trait(s) that best predict obesity associated health risk(s).

## Methods

Physical data and blood samples of 4000 subjects were collected between from 2012 to March- 2017 using a questionnaire and written informed consent was also signed by all the study participants. The study was approved by the Ethics committee of PirMehr Ali Shah Arid Agriculture University Rawalpindi (PMAS-AAUR) for the Use of Human Subjects (PMAS_AAUR/D. FoS/261).

The anthropometric data included height (feet-inches), body weight (Kg), blood pressure (mmHg), systolic (SBP) and diastolic (DBP) waist circumference (WC in cm), hip circumference (HC in cm), age, and gender of subjects were recorded.Body weight was recorded on a weighing balance without shoes and heavy clothing. Height was recorded while subjects were standing, without shoes, against a wall mounted scale. Waist circumference was measured with a measuring tap placed midway between lower rib margin and iliac crest while HC was measured as the maximum circumference over the buttocks. The biochemical analysis included lipid profile (TC, TG, HDL, LDL, and VLDL) and fasting blood sugar (BSF). All analyses were performed using serum on the spectrophotometer Microlab 300 (Merck). Obesity related indices like BMI, WHR, VAI, and BAI were calculated using standard formulas. Body mass index, ratio of weight (kg) and height, was calculated using formula (www.who.com):

$$\text{BMI}=\text{Weight}(\text{kg})/\text{Height}({\text{m}}^{2})$$

The WHR was calculated by dividing WC with HC..

For the calculation of VAI and BAI following sex specific formulas were used;

$$\mathrm{Male}:\mathrm{VAI}=\left(\frac{\mathrm{WC}}{39.68+(1.88\times \mathrm{BMI})}\right)\times \left(\frac{\mathrm{TG}}{1.03}\right)\times \left(\frac{1.31}{\mathrm{HDL}}\right)$$

$$\mathrm{Females}:\mathrm{VAI}=\left(\frac{\mathrm{WC}}{36.58+(1.89\times \mathrm{BMI})}\right)\times \left(\frac{\mathrm{TG}}{0.81}\right)\times \left(\frac{1.52}{\mathrm{HDL}}\right)$$

$$\mathrm{BAI}=\left(\frac{\mathrm{HC}}{{\left(\mathrm{HM}\right)}^{1.5}}\right)-18$$

Where,

BAI = Body Adiposity Index
HM = Height in Meters
HC = Hip Circumference in Centimeters

Metabolic risk phenotypes were recruited according to the criteria explained by International Diabetes Federation (IDF). According to IDF, WHR values >0.9 for men and >0.8 for women leads to the central obesity (waist circumference >94cm for men and >84 cm for women). According to IDF the risk of hyperglycemia increases as blood glucose levels raise >110mg/dl or 6.1mmol/L. Similarly abnormal lipid profile values including TG>1.70mmol/L, TC>5.18mmol/L, HDL<1.3mmol/L, and LDL>2.59mmol/L are predictor of dyslipidemia. Risk of hypertension was calculated on the bases of systolic and diastolic blood pressure as follows; >130mmHg systolic and >80mmHg for diastolic. All the samples were collected from population of Pakistan and subjects having pathogenic diseases were excluded from the study.

### Statistics

The study parameter were arranged into three groups; Anthropometric parameters, Biochemical profiles, Adiposity indices. Descriptive Statistics tests were used to compute means and standard deviations (SD) of all three study groups. The effect of BMI on study groups was estimated using linear regression (β values at 95% confidence intervals (CI)). The associations of adiposity indices with risk phenotypes (hypertension, dyslipidemia, hyperglycemia) in general as well as gender specific were computed using multinomial logistic regression with odds ratio (OR) at 95% confidence intervals (CI) with age and sex adjusted data. The significance of all statistical analysis was checked using a p≤0.05 (95%). All statistical analyses were performed using SPSS v. 21 (Chicago, IL, USA).

## Results

As presented in Table 1, the mean ranges of major anthropometric parameters such as WC, HC, SBP and DBP were found slightly higher than the normal ranges (Table 1). The anthropometric parameters, biochemical profiles, and adiposity indices showed highly significant associations with BMI (p≥0.0001). The effect size estimates (β at 95%CI) indicates that a unit increase in BMI (1 kg/m^2^) led to an increase in SBP/DBP, TC, TG, LDL, VLDL and 0.29 BSF but decrease in HDL levels (Table 2) was recorded. In case of adiposity indices highest relationship of BMI was found with BAI (β=0.76) followed by VAI (0.30) and WHR (0.27).

**Table 1:** Descriptive Characteristics of Study Variables

***Study Variables***	***Minimum***	***Maximum***	***Mean ± SD***
Age (yr)	20	65	35.34±11.15
Body Weight (Kg)	28	145	68.0±20.30
WC (cm)	25.4	215.90	89.75±19.95
HC (cm)	70	138	99.68±12.83
SBP (mmHg)	99	198	130.49±18.44
DBP (mmHg)	60	130	81.95±10.68
TC (mmol/L)	1.07	61.36	4.36±1.60
HDL (mmol/L)	0.014	3.6	1.28±0.48
LDL (mmol/L)	0.09	61.07	2.75±1.64
TG (mmol/L)	0.3	13.3	1.63±0.99
vLDL (mmol/L)	0.14	6.05	0.33±0.20
BSF (mmol/L)	2.3	35.944	5.90±2.25
WHR	0.83	1.11	0.93±0.03
BMI (Kg/m^2^)	16	62.48	25.05±7.31
VAI	0.049	48.185	1.64±2.40
BAI	17.8	48.313	28.25±6.22

BMI: Body mass index, WC: Waist circumference, SBP: Systolic blood pressure, DBP: Diastolic blood pressure, TC: Total cholesterol, HDL: High-density lipoprotein, LDL: Low-density lipoprotein, vLDL: Very low-density lipoprotein, TG: Triglycerides, FBG: Fasting blood glucose, BMI: Body mass index, WHR: Waist to Hip Ratio, VAI: Visceral adiposity Index, BAI: Basal adiposity index

**Table 2:** Effect size estimation of increase in BMI (per unit Kg/m^2^) on risk parameters

***Risk Parameters***	***Variables***	***β***	***CI 95%***	**P*-value***
Blood Pressure	Systolic (mmHg)	0.38	1.139–2.16	0.0006
	Diastolic (mmHg)	0.25	1.15–2.19	0.0007
Biochemical Profile	TC (mmol/L)	0.28	1.15–1.45	0.001
	TG (mmol/L)	0.29	1.9–2.5	0.0005
	LDL (mmol/L)	0.39	1.59–1.87	0.0026
	VLDL (mmol/L)	0.29	9.7–12.2	0.0001
	HDL (mmol/L)	−0.51	−8.24- (−7.35)	0.0006
	BSF (mmol/L)	0.29	0.04–0.52	0.0001
Obesity Indices	WC (cm)	0.74	2.5–3.7	<0.0001
	WHR	0.27	2.57–4.75	<0.0001
	VAI	0.30	1.01–1.82	<0.0001
	BAI	0.76	1.46–2.54	<0.0001

BMI: Body mass index, WC: Waist circumference, SBP: Systolic blood pressure, DBP: Diastolic blood pressure, TC: Total cholesterol, HDL: High-density lipoprotein, LDL: Low-density lipoprotein, vLDL: Very low-density lipoprotein, TG: Triglycerides, FBG: Fasting blood glucose, BMI: Body mass index, WHR: Waist to Hip Ratio, VAI: Visceral adiposity Index, BAI: Basal adiposity index

Association analysis of all adiposity indices with metabolic risk phenotypes is presented in Table 3. In our population, strongest association of BMI was found with hypertension (OR=10.2) followed by hyperglycemia (OR=4.7, CI=2.6–8.3) and dyslipidemia (OR=3.3, CI=1.1–10.1). Waist circumference showed highly significant association with hyperglycemia (OR=7.28) while a similar trend was seen in hypertension and dyslipidemia (OR=3.5) while WHR strongly correlated with hyperglycemia (OR=4.19) and hypertension (OR=2.13). In case of BAI strongest associations was found with hypertension (OR=7.8) and dyslipidemia (OR=5.2) while a mild one with hyperglycemia (OR=1.8). In case of VAI the magnitude of association with hyperglycemia and dyslipidemia were within similar ranges (OR=2.3) as compared to a lower correlation (OR=1.5) with hypertension. The results of gender specific associations of adiposity traits with risk phenotypes are presented in Table 4. All adiposity traits correlated significantly three risk phenotypes in both gender groups but the strongest associations in male population were of BAI with hypertension (OR=22.7) and hyperglycemia (OR=20.3). In comparison the females specific strongest association were found of BMI with hyperglycemia (OR=16.1), WC with hyperglycemia (OR=27.7) and dyslipidemia (OR=11.9), and BAI (OR=14.4).

**Table 3:** Association analysis of obesity indices with metabolic risk phenotypes

***Obesity Indices***	***Metabolic Risk Phenotypes***						
	***Hypertension***		***Hyperglycemia***		***Dyslipidemia***	
	***OR***	**P*-value***	***OR***	***p-value***	***OR***	**P*-value***
	(CI 95%)		(CI 95%)		(CI 95%)	
BMI	10.2 (6.3–16.6)	0.0001	4.7 (2.6–8.3)	0.0001	3.3 (1.1–10.1)	0.041
WC	3.5 (2.9–4.23)	0.0001	7.28 (5.7–9.28)	0.00001	3.5 (2.1–5.6)	0.00001
WHR	2.13 (1.2–3.9)	0.013	4.19 (1.14–6.6)	0.025	0.61 (0.19–1.9)	0.4
BAI	8.9 (5.6–14.2)	3.00E-04	1.8 (1.04–3.3)	0.037	5.2 (1.5–18.7)	0.01
VAI	2.3 (1.9–2.7)	0.0001	1.5 (1.2–1.8)	0.0001	2.3 (1.7–3.2)	0.0001

BMI: Body mass index, WHR: Waist to Hip Ratio, VAI: Visceral adiposity Index, BAI: Basal adiposity index

**Table 4:** Gender based association analysis of Obesity Indices with metabolic risk phenotypes

***Adiposity Traits***	***Metabolic Risk Phenotypes***												
	***Hypertension***				***Hyperglycemia***				***Dyslipidemia***			
	***Male***		***Female***		***Male***		***Female***		***Male***		***Female***	
	***OR (CI 95%)***	**P*-value***	***OR (CI 95%)***	**P*-value***	***OR (CI 95%)***	**P*-value***	***OR (CI 95%)***	**P*-value***	***OR (CI 95%)***	**P*-value***	***OR (CI 95%)***	**P*-value***
BMI	2.9 (1.99–4.1)	0.001	3.4 (1.9–6.01)	0.001	3.2 (2.3–4.5)	0.04	16.1 (8.9–19.2)	0.0001	6.7 (4.2–10.7)	0.01	2.9 (1.108.04)	0.03
WC	3.5 (2.8–4.42)	0.001	3.29 (2.3–4.71)	0.0001	5.8 (4.4–7.60)	0.001	27.7 (12.6–30.9)	0.000 1	2.86 (1.7–4.77)	0.01	11.9 (2.6–20.3)	0.001
WHR	1.6 (1.85–2.40)	0.009	3.9 (1.8–8.4)	0.0003	4.7 (1.9–11.9)	0.02	6.13 (1.6–23.3)	0.007	0.4 (0.03–3.9)	0.4	0.2 (0.05–0.9)	0.04
BAI	22.7 (8.7–11.8)	0.001	14.4 (4.7–17.6)	0.0001	20.3 (2.4–16.9)	0.005	3.7 (1.6–8.6)	0.002	3.07 (3.9–9.2)	0.005	3.22 (1.9–11.2)	0.016
VAI	2.89 (2.3–3.7)	0.001	3.5 (1.98– 6.7)	0.0001	1.3 (1.05–1.8)	0.02	1.5 (1.1–1.98)	0.03	3.08 (2.1–4.5)	0.001	4.01 (2.5–4.9)	0.005

BMI: Body mass index, WHR: Waist to Hip Ratio, VAI: Visceral adiposity Index, BAI: Basal adiposity index

## Discussion

Our study is the large scale population based data analysis that explored relationships of obesity indices; BMI, WC, WHR, BAI, and VAI with common metabolic health complications to identify a common risk marker of an obesogenic state. The BMI based effect size estimates clearly indicate that a unit increase in BMI significant raised all anthropometric and clinical risk markers. However, when association of all adiposity traits with hypertension, hyperglycemia and dyslipidemia were explored, the anthropometric marker WC maintained its strong yet significant correlations with all three metabolic risks in general as well as sex-specific adjusted population. The findings of this study clearly highlight WC as the surrogate marker of obesity associated health risks in local Pakistani populations. The highly significant associations of global anthropometric risk marker BMI were with hypertension and hyperglycemia in total population, which tended to vary when compared among males and females. However the role of BMI as a general obesity risk marker for Pakistani populations could not be underestimated based on its large effect sizes in relation to all clinical and anthropometric traits. Despite extensive global health awareness efforts, obesity has taken the form of an epidemic and predicted to be the one major cause of rapid rise in non-communicable disease (NCDs)(17). Obesity is now considered as the one most important risk factor for hypertension that may lead to the development of CVDs and chronic kidney disease even in children (18).We also report alarmingly high frequency of all major generalized obesity forms in our study population especially Class 3 (>40Kg/m^2^) with a rate of 15.8%. Likewise the frequencies of major metabolic risk phenotypes; hypertension, hyperglycemia and dyslipidemia in general population were; 33%, 21.9%, 9.4%. Thus a global rise in co-emergence of obesity and health complications does seem to indicate a strong link between them. However large variations in obesity phenotypes do seem to prevail in world populations ranging from general obesity to central/abdominal obesity as well their associated health consequences. Large inter-individuals variations have been observed with regards to the tendency to put on extra pounds either as general/abdominal obesity and associated health with strong ethnic influences (1). Therefore, internationally efforts are being made to employ simple yet affordable anthropometric scales (generalized, gender and age specific) for obesity measures as well as predictors of future health risks at large population scales (19). Many adiposity traits have been related to serious health issues (18) eventually leading to early death but these traits are not being extensively explored Worldwide (20).

The relationships among various anthropometric indices like BMI, WHR, WC, BAI, and VAI have been explored for the identification of specific obesity phenotypes and individual susceptibility towards disease risk (21). Among these indices, BMI is globally used for the measurement of general obesity while WC, HC and WHR are sex specific indices and are used for the measurement of central obesity (5). Our study results showed persistent increase in the levels of blood pressure, lipid profile (TC, TG, and LDL), BSF and a decrease in HDL with a unit increase in BMI. Thus BMI could serve as an excellent quantitative marker for the estimation of biochemical and anthropometric risks carried in general Pakistani populations carrying excess body weight. According to WHO criteria BMI could best describe relationship of obesity with cardiovascular or cardiometabolic risk factors (5). The highest health risk (OR=10.2) in our general population with raised BMI was hypertension followed by hyperglycemia (OR=4.7) while dyslipidemia associated weakly (OR=3.7, p=0.041). Our findings in accordance with previous studies conducted on Asians, Caucasians (22) and Maxicans(23) indicate higher risk of cardiometabolic diseases in individuals with raised BMI. Relationship of BMI with BAI, VAI, WC, and WHR was also explored with strong effects of raised BMI on basal adiposity index (Table 2). We found highly significant increases in BAI (β=0.76%) and WC (β=0.74%) with a unit increase in BMI. The relationship of BMI and BAI is being poorly studied, some studies reveal similar strength of BMI and BAI in predicting adiposity levels (24) while others suggest an inferior role of BAI as a marker for cardiometabolic diseases compared to BMI (12). Our results also clearly indicate BMI as an excellent general adiposity marker of local Pakistani populations.

On the other hand, strong associations of WC with major metabolic health complications in general and sex based population do seem to indicate a high risk of abdominal obesity in Pakistan (25). Thus our findings highlight the role of WC as a valuable marker of cardiometabolic risks for Pakistani populations regardless of sex. South Asian populations including Pakistan have been reported for higher susceptibility to abdominal weigh gain as compared to Europeans at a given BMI is reported (26)and development of advanced complications especially T2D, CVDs etc. (27). In parallel with WC, VAI also correlated strongly hypertension, hyperglycemia and dyslipidemia in general population though with slight variations when compared in male and females sub-groups. VAI is a gender-specific model derived from BMI, waist circumference, triglycerides and HDL-cholesterol from healthy normal/overweight adults (28). Previous studies have also indicated the role VAI as an indicator of obesity and associated health risks; hyperglycemia (29)MetS and CVDs (30). Though VAI estimates take into account both WC and lipid markers (TG and HDL), WC strongly predicted health risks in Pakistani populations participated in present study (Tables 2 and 3). Thus easy to measure, affordable, and non-invasive nature of WC estimates make it a perfect obesity marker for use at large population level to estimate health risks. Another good marker reported for obesity measures is WHR (31). We found statistically weak associations of WHR with hypertension and hyperglycemia in our population. In contrast to present study, previous findings rate WHR among best adiposity index for estimating the risk of CVDs (32). Though weak in total population groups, associations of WHR did improve slightly in our sex specific s (Table 3), raised WHR significantly increasing the risk of hypertension in female subjects. Likewise, BAI’s strong association in total population was only with hypertension which improved when compared among males and females.

## Conclusion

Based on highly strong associations, the present study results indicate BMI and WC as informative obesity markers that could be used for the prediction of metabolic risk factors in general Pakistani populations. Though large population was analyzed, our study could be considered as a pilot design that was aimed to explore relationships of anthropometric indices and adiposity traits with general forms of obesity focused to identify a common obesity marker that could best predict future health complications at large population scale. Our findings clearly highlight the current obesity scenario and associated health risks in Pakistan and thus could be adopted for the promotion of public health through lifestyle modifications with major focus on maintaining normal BMI and WC indices regardless of age, sex, and ethnicity.

## Ethical considerations

Ethical issues (Including plagiarism, informed consent, misconduct, data fabrication and/or falsification, double publication and/or submission, redundancy, etc.) have been completely observed by the authors.
